# Long-distance gene flow outweighs a century of local selection and prevents local adaptation in the Irish famine pathogen *Phytophthora infestans*

**DOI:** 10.1111/eva.12142

**Published:** 2014-02-18

**Authors:** Isabelle Glais, Josselin Montarry, Roselyne Corbière, Claudine Pasco, Bruno Marquer, Hélène Magalon, Didier Andrivon

**Affiliations:** 1 INRA, UMR1349 IGEPP (Institute of Genetics, Environment and Plant Protection)Le Rheu, France; † Laboratory of Marine Ecology, University of La RéunionSt Denis Messag Cedex 09, La Réunion, F-97715, France

**Keywords:** clonal lineage, evolution, gene flow, host resistance, local adaptation, microsatellites, potato late blight, selection

## Abstract

Sustainably managing plant resistance to epidemic pathogens implies controlling the genetic and demographic changes in pathogen populations faced with resistant hosts. Resistance management thus depends upon the dynamics of local adaptation, mainly driven by the balance between selection and gene flow. This dynamics is best investigated with populations from locally dominant hosts in islands with long histories of local selection. We used the unique case of the potato late blight pathosystem on Jersey, where a monoculture of potato cultivar ‘Jersey Royal’ has been in place for over a century. We also sampled populations from the coasts of Brittany and Normandy, as likely sources for gene flow. The isolation by distance pattern and the absence of genetic differentiation between Jersey and the closest French sites revealed gene flow at that spatial scale. Microsatellite allele frequencies revealed no evidence of recombination in the populations, but admixture of two genotypic clusters. No local adaptation in Jersey was detected from pathogenicity tests on Jersey Royal and on French cultivars. These data suggest that long-distance gene flow (∼ 50/100 km) prevents local adaptation in Jersey despite a century of local selection by a single host cultivar and emphasize the need for regional rather than local management of resistance gene deployment.

## Introduction

From an evolutionary ecology point of view, local adaptation (or maladaptation) between hosts and pathogens results from the balance between evolutionary forces acting simultaneously on pathogen life history traits involved in trade-offs (Gandon and Day 2009). Therefore, understanding the evolutionary mechanisms acting upon pathogen populations, and quantifying their balance, is essential to develop durable control strategies of these pathogens. This is the basis of Darwinian medicine in humans and animals (Nesse et al. 2010; Nesse 2011) and has the same applications for crop protection (Andrivon 2012).

However, predicting local adaptation remains difficult for both qualitative and quantitative pathogenicity traits. In the first case, corresponding to the boom-and-bust cycles observed in gene-for-gene systems, local adaptation is basically driven by disruptive selection following the appearance of virulent variants in an avirulent (*i.e*., incompatible) population, through mutation or gene flow (Kiyosawa et al. 1995; Brown and Tellier 2011). The virulent mutants are usually assumed to bear a fitness cost over avirulent genotypes on the hosts devoid of the matching resistance genes, although stable polymorphism can exist in the absence of such a cost (Leonard 1994; Tellier and Brown 2011). It has been possible to measure fitness costs of virulence in a few cases, although these costs are not always very high (Leach et al. 2001; Bahri et al. 2009; Montarry et al. 2010a), and can sometimes be compensated by additional mutations (Lannou 2012). Avirulent genotypes will therefore usually persist in the pathogen population as long as the selection pressure exerted by the hosts is low (*i.e.,* resistant hosts occupy a low fraction of the host acreage) or gene flow continuously brings into local populations genotypes maladapted to the local resistant hosts. This has been shown to occur in several *Blumeria graminis* populations in Europe (Andrivon and Limpert 1992). The same evolutionary processes probably apply also to quantitative resistance, but the complex nature of this type of resistance makes a prediction all the more difficult. Indeed, quantitative resistance often results from the alteration by the host of several pathogen life history traits, such as latent period duration, host colonization rate, or reproductive capacity (Clément et al. 2010). It is therefore polyphenic and polygenic, which makes an assessment of its fitness impact on pathogen (and host) genotypes both theoretically and experimentally challenging (Pariaud et al. 2009; Montarry et al. 2010b; Lannou 2012).

Theoretical models can be used to predict the occurrence of local adaptation (or lack thereof) in pathogen populations. They are based either on the comparisons of population differentiation for genetically neutral markers (*F*_ST_) and quantitative phenotypic traits (*Q*_ST_; Whitlock 2008) or on population genetics methods allowing to disentangle mean fitness estimates from the contribution of either gene flow and/or genetic drift (e.g., Whitlock and Gilbert 2012). Most of these models suggest that gene flow will tend to negate the selective effect and result in a lack of local adaptation (or sometimes even in local maladaptation) in the pathogens, although many specific circumstances, related either to the genetics of the traits (dominance, epistasis) or to the spatial and temporal distributions of host and pathogen populations can lead to apparent selection or to apparent neutrality on quantitative traits (Whitlock 2008; Blanquart et al. 2012).

Unfortunately, there is a general lack of suitable experimental data to validate these theoretical predictions over a range of situations. Such experimental data would be best collected in situations (i) favoring metapopulation dynamics (geographically close but distinct sites with frequent local extinction) and (ii) with different dominant host genotypes between sites. This conjunction is well met in the potato late blight pathosystem close to the English Channel. This pathosystem is indeed characterized by the following:

### A biotrophic pathogen

The oomycete *Phytophthora infestans* – feeding on an annual host. The strict dependence of the pathogen upon living host tissue to persist in the environment and the annual rather than perennial growth habit of its hosts make periodic local extinction highly likely. This assumption is confirmed by genetic data, which showed that over 90% of the genotypes present in local populations in the Netherlands disappear between one growing season and the next (Drenth et al. 1994);

### A fragmented host distribution

In western France, potato is commercially grown primarily in small coastal areas for the early table market. These spots are scattered along the northern coast of Brittany and of nearby Normandy. They are dedicated to vegetable production (typically potato, carrot, and leek in Normandy; potato, cauliflower, and artichoke in Brittany), with usually two different crops a year on each plot of land. This further restricts the vegetation period of each of them and therefore reinforces the chance for local extinction of biotrophic pathogens. Potato is a major crop in Jersey, one of the Channel Islands close to the coasts of Normandy and Brittany.

### Exposure to frequent and often strong winds

The oceanic situation and mid-latitude of this part of Europe goes along with a prevailing southwesterly wind regime, which may allow wind-borne spores to be dispersed easily over relatively large distances.

### Different dominant cultivars in Jersey and on the continent

In the French potato production area considered, cultivar Europa was dominant but not exclusive. By contrast, potato production on Jersey has been dedicated almost exclusively since the early 1900s to cv. Jersey Royal, a selection of the early cultivar International Kidney. Jersey Royal is grown year round in Jersey and is not produced anywhere else. Jersey therefore represents a unique situation in Europe, because of its island location, the extensive monoculture of a single potato cultivar for more than a century, and strict controls on movement of seed tubers of this cultivar into and out of Jersey (Deahl et al. 2009). Both cultivars are susceptible to *P. infestans,* according to the European Potato Database (http://www.europotato.org).

We used this unique situation to analyze experimentally the effect of gene flow and local selection on local adaptation in a host–pathogen system. Previous work showed that *P*. *infestans* populations from western France in 2004–2005 involved the admixture of two genetically distinct groups of clonal lineages (Montarry et al. 2010a), both adapted to the prevalent cultivar in Northwestern Europe, Bintje (Montarry et al. 2008). Here, we wanted to determine whether gene flow exists between populations in Jersey and on the continent and would be sufficient to negate the effect of extensive, prolonged local selection by a single host cultivar, Jersey Royal. To this end, we tested the hypothesis of local adaptation to Jersey Royal in the *P. infestans* population present in Jersey, using both genotypic and phenotypic data from isolates sampled in Jersey and in four distinct French potato-cropping areas during a single epidemic and a local adaptation cross-inoculation experiment.

## Materials and methods

### Collecting and maintaining *Phytophthora infestans* isolates

During the summer of 2006, 232 *P. infestans* isolates were collected from 19 potato fields distributed across five sites in Jersey, Brittany (Ploudaniel, Paimpol and Saint-Malo), and Normandy (Val de Saire; Table 1). Fields within a site were separated by 1–10 km. At each site, samples were taken from the locally dominant cultivar: Jersey Royal in Jersey, Bintje in Ploudaniel (western Brittany), and Europa in the three other French sites (Paimpol, Saint-Malo and Val de Saire). Plants were haphazardly sampled across each field, and only one leaf per plant was sampled. Single lesion isolates were established and maintained as axenic cultures on pea agar medium as previously described (Montarry et al. 2010a). Isolates were kept at 15°C in the dark and transferred every 4–6 weeks to fresh agar media until phenotypic characterization or DNA extraction.

**Table 1:** Sources of isolates of *Phytophthora infestans* collected in summer 2006.

Location	Potato cultivar	Sampling date	No. samples (No. fields)	A1/A2	GPS central point
Jersey	Jersey Royal	15/05/2006	48 (5 fields)	48/0	49°13′02.25″N 2°04′27.93″O
Paimpol	Europa	02/06/2006	46 (5 fields)	33/13	48°46′35.89″N 3°04′34.95″O
Saint-Malo	Europa	22/06/2006	51 (2 fields)	51/0	48°37′0.45″N 1°57′21;36″O
Ploudaniel	Bintje	06/07/2006	41 (3 fields)	40/1	48°30′06.10″N 4°19,27.47″O
Val de Saire	Europa	12/07/2006	46 (4 fields)	46/0	49°39′21.05″N 1°16′38.44″O

### Mating type determination

*Phytophthora infestans* is a heterothallic species, with two mating types named A1 and A2. The production of sexual offspring (oospores) occurs only when A1 and A2 isolates come into contact. The mating type of each isolate was identified by pairing it on pea agar medium with known A1 and A2 tester isolates, incubating in the dark at 15°C for 10–14 days, and looking under the microscope for the presence of oospores where the two colonies met. Isolates producing oospores with the A1 tester were rated as A2 mating type and vice versa.

### Genotyping isolates

Isolates were grown in pea broth, previously autoclaved for 20 min at 120°C and cooled to room temperature. After 10–15 days of incubation at 15°C, mycelium was washed three times in sterile water, dried on sterile paper, and lyophilized. DNA was extracted using the NucleoSpin® Tissue kit (Macherey-Nagel GmbH and Co, Düren, Germany), according to the manufacturer's instructions. DNA concentration and purity were estimated for some isolates using a spectrofluorimeter Nanodrop ® ND-1000 (Fisher Scientific, Illkirch, France).

Fourteen polymorphic SSR loci: Pi02, Pi 04, Pi16, Pi33, Pi56, Pi63, Pi70, Pi89 (Lees et al. 2006), D13, G11, 4B, 4G (Knapova and Gisi 2002), M19, and M34 (developed in our laboratory) were amplified using polymerase chain reaction (PCR). For M19 and M34, respectively, the forward primers were 5′-cacgacgttgtaaaacgactagtggagacgttgtgcagg-3′ and 5′-cacgacgttgtaaaacgaccctttagcagctcatttccg-3′, and the reverse primers were 5′-gtggctgcggatacttcttt-3′ and 5′-cgcactcgtcttgttgcac-3′. All PCRs were performed in a 12.5 *μ*L volume, as described by Montarry et al. (2010b). To detect simultaneously the alleles at several loci, the primers used were labeled with four fluorescent dyes: FAM (D13, Pi63, Pi02, Pi70, and M19), NED (G11, 4G, Pi89, Pi33, and M34), VIC (Pi04 and Pi16), and PET (Pi56 and 4B). Nonlabeled primers were provided by Sigma-Genosys Ltd (Sigma-Aldrich, St. Louis, MO, USA), and labeled primers by Applied Biosystems UK (Warrington, Cheshire, UK). The PCRs were performed in a MJ Research PTC-225 thermocycler under the following conditions: one cycle of 5 min at 94°C, followed by 30 cycles of 20 s at 94°C, 25 s at 58°C, and 30 s at 72°C, and a final elongation cycle of 5 min at 72°C.

Amplification products were pooled into four batches, based on expected allele sizes: batch1, Pi02, Pi89, and 4B; batch 2, G11, Pi04, Pi56, Pi63, and Pi70; batch 3, D13, 4G, Pi16, and Pi33; batch 4, M19 and M34. For each batch and isolate, a 10-*μ*L sample comprising 9.84 *μ*L deionized formamide Hi-Di™ (Applied Biosystems), 0.06 *μ*L of 400 HD ROX™ size standards (Applied Biosystems), and 0.1 *μ*L of PCR multiplexed products were loaded into an ABI Prism 3130*xl* DNA Sequencer (Applied Biosystems) and run according to the manufacturer's instructions. DNA fragments were automatically sized with GeneMapper™ v4.0, and allele sizes were calibrated to the allele sizes of reference isolates (C1–C12) kindly provided by Drs A.K. Lees and D.E.L Cooke (James Hutton Institute, Dundee, UK).

### Quantitative pathogenicity

Plants of the potato cultivars Bintje, Europa, and Jersey Royal were grown from certified seed tubers in a glasshouse regulated at 15–20°C (night/day temperatures) and 16 h of photoperiod, in 13-cm pots (one tuber per pot) filled with sand–peat–compost mixture. Plants were watered with a nutrient solution (Hakaphos; NPK 15/10/15) once a week. Leaflets were collected on 6-to 8-week-old plants. All experiments were performed from mid-September to mid-December 2007.

To restore pathogenicity possibly lost during axenic cultures, each isolate was first multiplied separately on detached leaflets of the susceptible cultivar Bintje. Each leaflet was placed abaxial side up and infected by deposing a drop of a suspension of *P. infestans* sporangia, prepared by flooding a 3-week-old culture with 5 mL sterile distilled water and gently scrapping the colony surface with a small glass rod. After 7 days of incubation in humid chambers under controlled conditions (15°C/18°C night/day temperatures, 16-h daylight), the new sporangia produced on the infected leaflets were collected in sterile water, and the concentrations of the resulting suspensions were adjusted to 5 × 10^4^ sporangia mL^−1^. Suspensions were kept at 4°C for approximately 2–3 h to promote zoospore release, just before being used for pathogenicity experiments (Montarry et al. 2008).

Each isolate was tested for lesion size and spore production on all three host cultivars (Bintje, Europa, and Jersey Royal). Six leaflets per cultivar × isolate combination were placed abaxial face up on the lids of inverted Petri dishes containing 10 g L^−1^ water agar (two leaflets per dish) and infected by depositing a 20 *μ*L drop of the calibrated sporangial suspension at the leaflet center. Infected leaflets were incubated for 6 days as described above. Lesion diameters were measured in two perpendicular directions, and lesion size was computed assuming an elliptic shape. Then, each leaflet was washed in 10 mL saline buffer (Isoton II), and sporangia were counted with a Beckman Coulter Z2 cell counter (Beckman, Villepinte, France) to determine sporangial production per lesion.

### Data analyses

#### Genetic data

Among the fourteen microsatellite loci, only twelve were analyzed because over 15% of the isolates showed multiple banding at loci M19 (28 isolates showing three bands and five showing four bands) and Pi63 (all isolates showing three bands), casting doubt as to the actual ploidy level at these loci. Moreover, to have a correct assignment to multilocus genotypes, all isolates with amplification in which amplification failed for at least one locus were excluded from the final data set, which therefore included 179 *P. infestans* isolates without any missing data.

Repeated multilocus genotypes (MLGs), that is, isolates sharing the same alleles at all 12 loci, and the number of copies of each MLG (*n*) were identified using GenClone 2.0 (Arnaud-Haond and Belkhir 2007). Genotypic diversity was estimated as the genotypic richness R  =  (number of different MLGs – 1)/(total number of isolates − 1). Because an MLG may result from distinct sexual reproduction events or from clonal reproduction, we estimated the probability that the n occurrences of a given MLG results from sexual reproduction, given the observed allele frequencies for each population (Psex n re-encounter). Because the inclusion of clonal multicopies can strongly distort the *F*-statistics, we considered only one copy of each multilocus genotype for all subsequent genetic analyses (*i.e.,* a clone-corrected data set). An exception is the computation of the genetic diversity indices, for which statistics were performed both with and without clonal copies.

The evolutionary relationships among *P. infestans* MLGs were inferred from a minimum spanning network, calculated using minspnet (Excoffier and Smouse 1994) from the matrix of genetic distances based on the number of different alleles (DAS), and represented graphically *via* the neato layout engine using graphviz (http://graphviz.org).

Linkage disequilibrium between loci was calculated with genepop (with default Markov chain parameters) to count the number of locus pairs showing significant linkage disequilibrium across all populations. A Bonferroni correction (adjusted α = 0.0009; 55 comparisons) was applied to take into account multiple testing.

Genetic diversity in each population (and each cluster) was estimated as both the allelic richness (Ar) and gene diversity. Allelic richness was estimated using the rarefaction method implemented in populations 1.2.30 (Langella 1999), which estimated the mean number of alleles per locus for a reduced sample size. An unbiased estimate of gene diversity (H_E_ according to Nei, 1978) and deviation from random mating (*F*_IS_) were computed using genetix 4.05.2 (Belkhir et al. 1996). The statistical significance of *F*_IS_ values for each population (and each cluster) was tested using the resampling method (1000 bootstraps) implemented in genetix.

The differentiation coefficients between each pair of *P. infestans* populations (*F*_ST_) were estimated according to Weir and Cockerham (1984) using genepop 4.0.7 (Raymond and Rousset 1995), and their statistical significance was tested with the exact *G*-test (with default Markov chain parameters) implemented in genepop. Following the recommendations of Rousset (1997), the hypothesis of isolation by distance was tested by calculating the correlation between the matrices representing pairwise Slatkin genetic distances *F*_ST_/(1−*F*_ST_; Slatkin 1995) and the natural logarithm of geographic distance for each pair of *P. infestans* populations. The statistical significance of the correlation was assessed with a Mantel test (10 000 permutations) using xlstat 2012 (Addinsoft SARL, Paris, France).

Without taking into account their geographic origin, *P. infestans* isolates were clustered on the basis of their genetic relatedness using the Bayesian clustering approach implemented in structure 2.3.3 (Pritchard et al. 2000; Falush et al. 2003). Simulations were performed using the admixture model. We estimated the number K of genetic clusters (here between K = 1 and K = 15) to which the isolates should be assigned. For all simulations, we did not force the model with predefined allele frequencies for source clusters. Five independent runs were conducted to assess the consistency of the results across runs, and all runs were based on 500 000 iterations after a burn-in period of 100 000 iterations. We then identified the number of genetically homogeneous clusters as described by Evanno et al. (2005).

#### Phenotypic data

Normality and homogeneity of variances were checked with the Shapiro–Wilk and the Leven tests, respectively, using the statistical software R, version 2.15.0 (The R Foundation for Statistical Computing, 2012). The effects of potato cultivar, *P. infestans* population, and the corresponding interaction on lesion size and spore production were tested through an anova model using the statistical software R. A second anova model was used to test the *P. infestans* population effects separately for each potato cultivar. Indeed, when environments are defined as different host resistance levels, the ‘home versus away’ criterion is poorly informative regarding adaptation to the host, because it is biased by an important host effect, and the relevant test for local adaptation becomes the ‘local versus foreign’ criterion (Thrall et al. 2002; Kawecki and Ebert 2004; Montarry et al. 2008). When significant effects were detected, mean values were compared with Tukey's HSD tests (*α *= 0.05).

## Results

### Mating type

Most isolates belonged to the A1 mating type: only 14 isolates of the 232 isolates were A2 and were found in one field sample at Paimpol (13 isolates) and one isolate in Ploudaniel (Table 1). Among those 14 A2 isolates, 12 isolates, all from Paimpol, were successfully genotyped.

### Multilocus genotype analysis

Forty-four alleles could be amplified across the 12 microsatellite loci, with two to seven alleles per locus. GenClone identified 23 unique multilocus genotypes (MLGs) among the 179 *P. infestans* isolates, corresponding to a genetic richness of *R* = 0.128. Fifteen MLGs were represented by a single isolate (Table 2). Three of the eight repeated MLGs were found in at least two populations: (i) MLG9 included four isolates from Paimpol and nine isolates from Ploudaniel; (ii) MLG16 included 15 isolates from Jersey, seven isolates from Paimpol, 10 isolates from Saint-Malo and four isolates from Val de Saire; and (iii) MLG18 included 19 isolates from Jersey, 12 isolates from Paimpol, 20 isolates from Saint-Malo, 11 isolates from Ploudaniel, and 31 isolates from Val de Saire. The very low *P*_sex_ n re-encounter values indicated that the overrepresentation of these repeated MLGs probably results from clonal amplification (Table 2).

**Table 2:** Characteristics of the 23 multilocus genotypes (MLGs) which were discriminated using 12 microsatellite loci on the 179 *Phytophthora infestans* isolates. Cluster assignment, number of copies (*n*), mating type, and *P*_sex_ n re-encounter are indicated for each MLG.

MLG	Cluster assignment	*n*	Mating type	*P*_sex_ n re-encounter
MLG18	1	93	A1	2.67E-256
MLG16	1	36	A1	6.50E-110
MLG12	1	6	A1	2.31E-15
MLG21	1	2	A1	4.18E-05
MLG22	1	2	A1	1.53E-06
MLG5	1	1	A1	
MLG6	1	1	A1	
MLG11	1	1	A1	
MLG 13	1	1	A1	
MLG 14	1	1	A1	
MLG 15	1	1	A1	
MLG 17	1	1	A1	
MLG 19	1	1	A1	
MLG20	1	1	A1	
MLG23	1	1	A1	
MLG9	2	13	A1	2.10E-84
MLG2	2	9	A2	4.24E-48
MLG1	2	3	A2	2.25E-13
MLG3	2	1	A1	
MLG4	2	1	A1	
MLG7	2	1	A1	
MLG8	2	1	A1	
MLG10	2	1	A1	

Two MLGs (MLG1 and MLG2) included only isolates belonging to the A2 mating type (Table 2). Comparing their fingerprints with those described by Cooke et al. (2012) showed that they differed markedly from the dominant MLGs in Great Britain at the time, that is, 13_A2, and were rather variants of MLG 3_A2, a rapidly declining lineage in the British Isles between 2005 and 2007. A similar observation was made regarding the main A1 MLGs, which were variants of either 1_A1 (MLG9) or 2_A1 (MLG12,-16, and-18) rather than of either 6_A1 or 8_A1, which were the major A1 lineages in Britain at the time. MLG18, which is the most abundant within our samples, is also similar to MLG JE-1.5 reported in Jersey during the same period (Deahl et al. 2009).

### Genetic structure of *Phytophthora infestans* populations

Four to nine MLGs were found in each of the five populations, the population from Ploudaniel showing the highest genotypic diversity (*R* = 0.30; Table 3). Whatever the data set used (with or without clonal copies), the mean allelic richness (Ar) per population ranged from 1.88 to 2.41 and gene diversity (*H*_E_) from 0.383 to 0.514 (Table 3). The highest Ar and H_E_ were found in populations from Paimpol and Ploudaniel. *F*_IS_ estimates for each population were in the majority of cases significantly negative, ranging from −0.769 to −0.151 and from −0.645 to 0.018 for data sets with and without clonal copies, respectively (Table 3). Moreover, 12 locus pairs among the 55 possible comparisons showed significant linkage disequilibrium (*P *<* *0.05) across all populations if no corrections for multiple tests across all populations were made, although none showed significant linkage disequilibrium after Bonferroni's correction.

**Table 3:** Genotypic and genetic diversity indices for each *Phytophthora infestans* population (A) and for each cluster (B). N, number of isolates; MLG, number of distinct multilocus genotypes; R, genotypic richness (MLG-1)/(N-1); H_E_, unbiased estimate of gene diversity (Nei, 1978); Ar, allelic richness corrected for sample size (*n*); *F*_IS_ significantly different to zero are indicated in bold. The genetic statistics (H_E_, Ar, and *F*_IS_) were performed both with and without clonal copies.

				Genetic diversity					
	Genotypic diversity			With clonal copies			Without clonal copies		
A	*N*	MLG	*R*	*H*e	Ar (*n* = 28)	*F*is	He	Ar (*n* = 4)	*F*is
Jersey	36	4	0.09	0.384	1.88	−0.765	0.470	1.92	−0.645
Paimpol	36	6	0.14	0.462	2.41	−0.369	0.514	2.33	−0.212
Saint-Malo	38	5	0.11	0.383	1.91	−0.757	0.456	1.91	−0.553
Ploudaniel	28	9	0.30	0.433	2.33	−0.151	0.443	2.19	0.018
Val de Saire	41	7	0.15	0.378	1.95	−0.769	0.449	1.93	−0.486
				Genetic diversity					
	Genotypic diversity			With clonal copies			Without clonal copies		
B	*N*	MLG	*R*	*H*e	Ar (*n* = 30)	*F*is	He	Ar (*n* = 4)	*F*is
Cluster 1	149	15	0.09	0.381	2.06	−0.751	0.459	2.25	−0.451
Cluster 2	30	8	0.24	0.333	2.08	−0.422	0.344	2.08	−0.264

**Figure 1:**
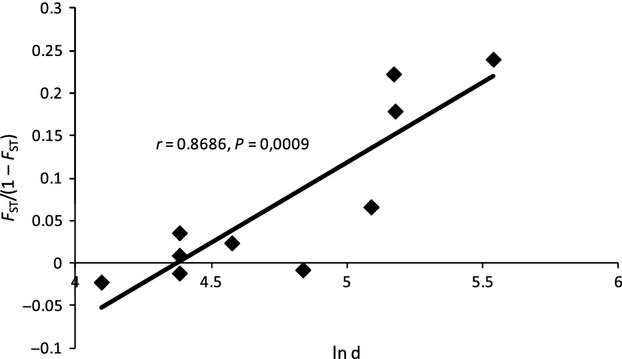
Isolation by distance pattern between genetic differentiation, measured as *F*_ST_/(1 − *F*_ST_), and geographic distance (natural logarithm of the distance in km) for pairwise *Phytophthora infestans* populations.

The genetic differentiation between populations was significant in four of the ten pairwise *F*_ST_ values (Table 4). The population from Ploudaniel was significantly highly differentiated (*P* < 0.01; Table 4) from those from Val de Saire (*F*_ST_ = 0.194), Saint-Malo (*F*_ST_ = 0.182), and Jersey (*F*_ST_ = 0.152). To a lesser extent, populations from Paimpol and Val de Saire were also genetically differentiated (*F*_ST_ = 0.062). Consequently, a significant correlation was observed between geographic distance and *P. infestans* population differentiation (Mantel test, *n* = 9; Pearson's coefficient of correlation *r* = 0.87; *P* = 0.0009; Fig. 1). The isolation by distance pattern was, however, nonsignificant when the population from Ploudaniel was excluded (*r* = 0.65, *P* = 0.164), consistent with the low differentiation among the easternmost populations sampled (Table 4, Fig. 3).

**Table 4:** Pairwise *F*_ST_ distances between *Phytophthora infestans* populations.

	Jersey	Val deSaire	Saint-Malo	Paimpol
Val deSaire	−0.012			
Saint-Malo	−0.023	−0.008		
Paimpol	0.009	0.062*	0.035	
Ploudaniel	0.152**	0.194***	0.182***	0.023

Significance of *F*_ST_ is indicated by stars

* (<0.05,

** <0.01 and

*** <0.001).

### Clustering analysis and geographic repartition of genetic clusters

**Figure 2:**
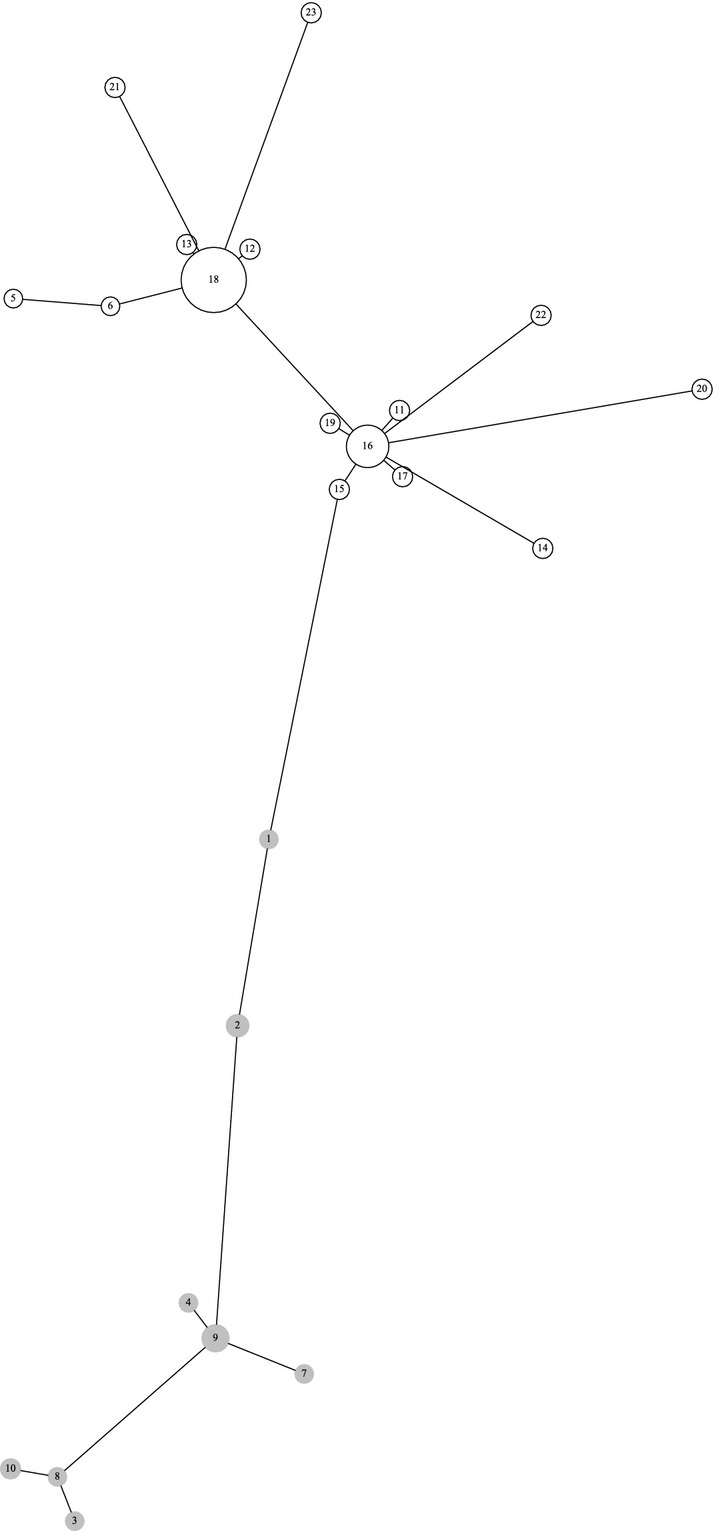
Minimum-spanning network showing the relationships among the *Phytophthora infestans* multilocus genotypes (MLGs 1 to 23) of both clusters (1 in white and 2 in gray). Branch sizes are proportional to genetic distance (*i.e.,* the number of different alleles) and circle areas to the numbers of isolates.

A Bayesian clustering analysis was performed using structure on the clone-corrected data set (*i.e.,* 23 *P. infestans* MLGs). That clustering analysis clearly indicated that the posterior distribution of the allele frequencies among clusters was best explained with a grouping into *K* = 2 genetic clusters. Applying an assignment threshold of 0.90, 91% of the MLGs (21 of 23) could be assigned to one or the other genetic cluster, confirming that the two clusters were well differentiated (*F*_ST_ = 0.317). The evolutionary relationships among the *P. infestans* MLGs from both genetic clusters were showed by a minimum spanning network (Fig. 2).

**Figure 3:**
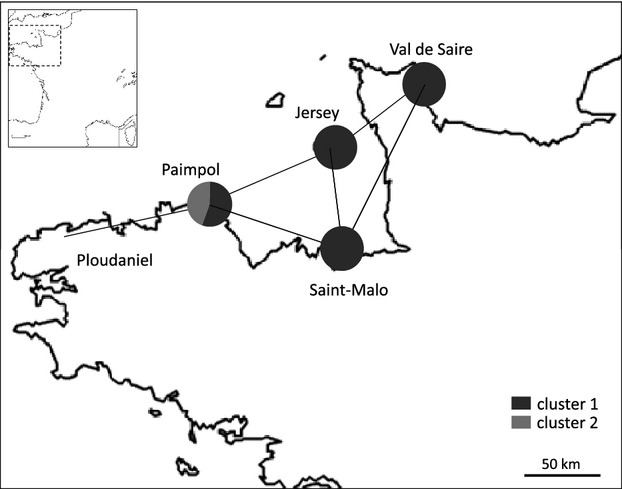
Geographic position of each *Phytophthora infestans* population used in this study and mean assignment percentage for each population to each genetic clusters. The populations linked by black lines are not genetically differentiated (*P *>* *0.05) based on an *F*_ST_ test, and those not linked by black lines are genetically differentiated (*P *<* *0.05).

Cluster 2 had a higher genotypic diversity (*R* = 0.24 with eight MLGs for 30 isolates) than cluster 1 (*R* = 0.09 with 15 MLGs for 149 isolates; Table 3). Although cluster 1 included only isolates of the A1 mating type, whereas cluster 2 included both mating types (A1 and A2), H_E_ and Ar were higher for cluster 1 than for cluster 2, especially in analyses performed without clonal copies (Table 3). Both clusters showed significantly negative *F*_IS_ (Table 3). While cluster 1 included isolates from all populations, only isolates from Ploudaniel and Paimpol were present in cluster 2 (Fig. 3).

### Pathogenicity of *Phytophthora infestans* populations on different cultivars

**Figure 4:**
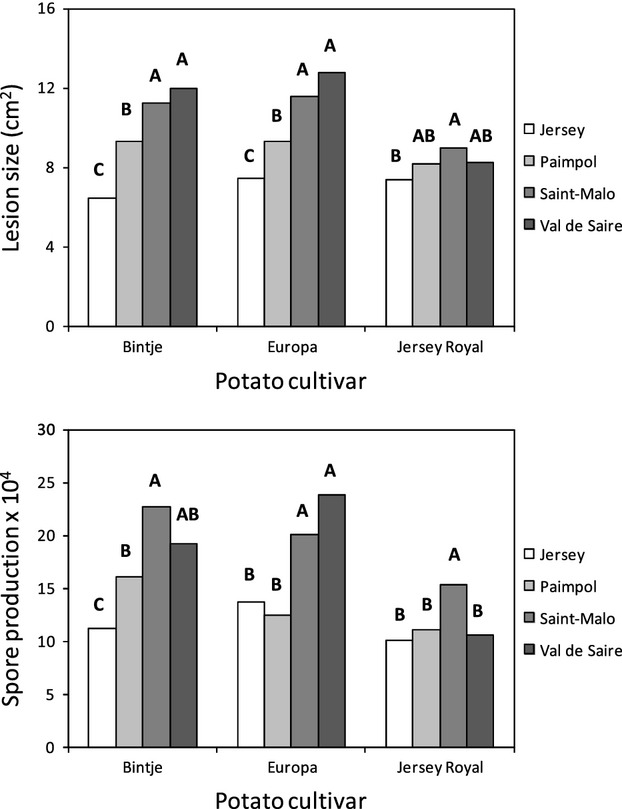
Mean lesion size and spore production of each *Phytophthora infestans* population, from Jersey, Paimpol, Saint-Malo, and Val de Saire, on the different potato cultivars, Bintje, Europa, and Jersey Royal. Letters represent the homogenous groups identified with the Tukey's HSD test at the 5% threshold.

All tested explanatory factors (*i.e*., *P. infestans* populations, potato cultivars, and their interaction) had a significant effect on both lesion size and spore production (Table 5). Overall, Jersey Royal cv. tended to be less susceptible than the other two cultivars (Fig. 4), and the population from Jersey tended to be less pathogenic than the French ones, irrespective of the cultivar tested (Fig. 4), explaining the significant population effect in the anova (Table 5) and suggesting general maladaptation in this population. The second anova model showed significant *P. infestans* population effects for each potato cultivar (Bintje, Europa, and Jersey Royal) and each pathogenicity trait (lesion size and spore production), except for lesion size on Jersey Royal where the population effect was only marginally significant (*F*_3,157_ = 2.54, *P* = 0.058).

**Table 5:** Anova of lesion size and of spore production. Sources of variation are *Phytophthora infestans* population, potato cultivar, and the corresponding two-way interaction between these variables.

	Lesion size				Spore production			
Source of variation	df	Mean square	*F* value	*P* > *F*	df	Mean square	*F* value	*P* > *F*
*Phytophthora* infestans population	3	1212.6	51.89	<0.0001***	3	5.0E + 11	28.17	<0.0001***
Potato cultivar	2	318.7	20.46	<0.0001***	2	2.9E + 11	24.82	<0.0001***
Population * cultivar	6	308.2	6.59	<0.0001***	6	1.9E + 11	5.51	<0.0001***
Error	478	3723.7			472	2.8E + 12		

Statistically significant effects are indicated by asterisks

* (*P* < 0.05;

** *P* < 0.01;

*** , *P* < 0.001); df, degrees of freedom.

## Discussion

Earlier work with geographically distant populations has shown that local adaptation can emerge in *P. infestans* (Andrivon et al. 2007), but is negated when populations are strongly connected and/or submitted to largely uniform selection pressures by a single host cultivar (Montarry et al. 2008). Here, we expected local adaptation to be detectable in Jersey, because of its insularity and of prolonged selection by a host cultivar specific to this island. Furthermore, the mild climate and year-round presence of the host were expected to limit the extinction probability of locally adapted genotypes and therefore to reinforce such a local adaptation pattern.

However, our data revealed no pattern of local adaptation to cv. Jersey Royal in Jersey, and extensive gene flow between *P. infestans* populations in Jersey and in the neighboring parts of France. The low genetic differentiation between the population from Jersey and its three nearest neighbors from France (Val de Saire, Saint-Malo and Paimpol) supports the hypothesis of intensive gene flow at this scale, despite the apparent phenotypic differences in pathogenicity. Together, these observations strongly suggest that gene flow between France and Jersey is preventing the evolution of local adaptation patterns, despite persistent and widespread selection in Jersey over more than a century by a single host cultivar specific to this island.

The geographic differentiation between local populations gives clues as to the effective distance upon which gene flow is active in this pathosystem. This distance is in the order of 100–120 km, according to the *F*_ST_ calculations between pairs of populations. It explains the significant isolation by distance pattern when the Ploudaniel population is considered, but the lack of significance of this pattern is explained when this population, further west, is excluded from the data set. The dispersal range calculated here markedly exceeds the usual assumptions, of the order of magnitude of a few kms, regarding the effective dispersal kernels of the short-lived, fragile sporangia of *P. infestans* (see Skelsey et al. 2010). This may be due to the very conducive climatic conditions favoring dispersal of the pathogen in western France, with strong winds, widespread potato crops, and frequent episodes of high humidity, which all favor infection by disseminated spores.

A further factor favoring effective dispersal and general adaptation is the genetic proximity between hosts. Searching the European Potato Database (http://www.europotato.org) reveals that cv. Europa has cv. Bintje among its progenitors, through cvs. Climax and Edzina. It is therefore understandable that adaptation to cv. Bintje, previously shown in French populations (Montarry et al. 2008), might also confer adaptation to some of its offspring. This then favors the persistence of a large population with the same core adaptive features and thus limits the possibility for emergence of local adaptation patterns and metapopulation dynamics with divergent selection histories.

Our finding that *P. infestans* populations cluster into two distinct genetic groups is consistent with previous data from French isolates collected in 2004 and 2005 (Montarry et al. 2010b) revealing that the admixture pattern was stable over at least three successive years (from 2004 to 2006). The maintenance of the differentiation between those clusters could be explained by the fact that sexual reproduction seems to play no role in the adaptive patterns recorded here. The population structures observed remained highly clonal, although admixed, and no sign of recombination was observed despite the co-occurrence of both mating types in two of the populations surveyed (Paimpol and Ploudaniel). Indeed, the *P*_sex_ values and the negative *F*_IS_ values, indicating an excess of heterozygotes that could result from asexual reproduction (Halkett et al. 2005; Goyeau et al. 2007), bring strong evidence for clonal reproduction in these populations. This general structure, consistent with earlier observations (Montarry et al. 2010b), probably further strengthens the persistence of general adaptation to Bintje rather than the emergence of local adaptation.

These observations have major consequences for resistance deployment strategies and the management of late blight epidemics through resistant cultivars. Such deployment strategies, to be effective, should ideally exploit local maladaptation generated when the hosts are diverse and the pathogen moves rapidly (Kawecki and Ebert 2004). They can also rely on a strong metapopulation dynamics resulting from limited gene flow and frequent local extinction. The strictly biotrophic nature of *P. infestans* and its host range limited to a few annual hosts should favor such local extinction. However, the widespread presence of potato crops throughout the territory and the extensive gene flow shown here imply that a metapopulation dynamics is quite unlikely to take place. This is consistent with population structures of *P. infestans* in western Europe being dominated by a few, widely distributed clonal lineages, such as US-1 until the early 1980s (Spielman et al. 1991) or 13_A2 recently (Cooke et al. 2012).

Therefore, it will probably be difficult to think of effective resistance deployment strategies over large scales, such as crop mosaics or refuge areas, because the strong gene flow and connection of host populations will make it hard to generate sufficient differentiation in local parasite populations for a large-scale spatial deployment strategy of resistant hosts to be effective (Skelsey et al. 2010). The use of highly differentiated host genetic backgrounds, for instance the introduction of different resistant R gene pyramids through *ad-hoc* cassettes in a cisgenesis transformation process (Haverkort et al. 2008), might help, although the durability of gene pyramids has proven low in the past due to the large effective population sizes of the pathogen (e.g., Andrivon 1994). A better option might thus be to promote within-crop resistance diversification through cultivar or species mixtures (Garrett and Mundt 2000; Andrivon et al. 2003; Philips et al. 2005; Bouws and Finckh 2008), which minimizes genotype unit area and has proven effective when disease pressure can be maintained at a moderate level (Pilet et al. 2006), rather than diversification strategies targeted at the landscape or regional spatial scales.

## Data archiving statement

Data for this study are available from the Dryad Digital Repository: http://dx.doi.org/10.5061/dryad.ch200.

## References

[b1] Andrivon D (1994). Race structure and dynamics in populations of *Phytophthora infestans*. Canadian Journal of Botany.

[b2] Andrivon D (2012). Dynamics of change in human-driven and natural systems: fast forward, slow motion, same movie? A case study from plant protection. Sustainability.

[b3] Andrivon D, Limpert E (1992). Origin and proportions of the components of composite populations of *Erysiphe graminis* f. sp. *hordei*. Journal of Phytopathology.

[b4] Andrivon D, Lucas JM, Ellissèche D (2003). Development of natural late blight epidemics in pure and mixed plots of potato cultivars with different levels of partial resistance. Plant Pathology.

[b5] Andrivon D, Pilet F, Montarry J, Hafidi M, Corbière R, Achbani EH, Pellé R (2007). Adaptation of *Phytophthora infestans* to partial resistance in potato: evidence from French and Moroccan populations. Phytopathology.

[b6] Arnaud-Haond S, Belkhir K (2007). GENCLONE: a computer program to analyse genotypic data, test for clonality and describe spatial clonal organization. Molecular Ecology Notes.

[b7] Bahri B, Kaltz O, Leconte M, Enjalbert C, Vallavieille-Pope de J (2009). Tracking costs of virulence in natural populations of the wheat pathogen, *Puccinia striiformis* f. sp. *tritici*. BMC Evolutionary Biology.

[b8] Belkhir K, Borsa P, Chikhi L, Raufaste N, Bonhomme F (1996). GENETIX 4.05, Logiciel Sous Windows TM Pour La Génétique Des Populations.

[b9] Blanquart F, Gandon S, Nuismer SL (2012). The effect of migration and drift on local adaptation to a heterogeneous environment. Journal of Evolutionary Biology.

[b10] Bouws H, Finckh MR (2008). Effects of strip intercropping of potatoes with non-hosts on late blight severity and tuber yield in organic production. Plant Pathology.

[b11] Brown JKM, Tellier A (2011). Plant-parasite coevolution: bridging the gap between genetics and ecology. Annual Review of Phytopathology.

[b12] Clément JAJ, Magalon H, Pellé R, Marquer B, Andrivon D (2010). Alteration of pathogenicity-linked life-history traits by resistance of its host *Solanum tuberosum* impacts sexual reproduction of the plant pathogenic oomycete *Phytophthora infestans*. Journal of Evolutionary Biology.

[b13] Cooke DEL, Cano LM, Raffaele S, Bain RA, Cooke LR, Etherington GJ, Deahl KL (2012). Genome analyses of an aggressive and invasive lineage of the Irish potato famine pathogen. PLoS Pathogens.

[b14] Deahl KL, Perez FM, Thompson JM, Fleming-Archibald C, Thompson S, Collier R, Kildea S (2009). Characterization of *Phytophthora infestans* isolates from Jersey, Channel Islands. Potato Research.

[b15] Drenth A, Tas ICQ, Govers F (1994). DNA fingerprinting uncovers a new sexually reproducing population of *Phytophthora infestans* in the Netherlands. European Journal of Plant Pathology.

[b16] Evanno G, Regnaut S, Goudet J (2005). Detecting the number of clusters of individuals using the software STRUCTURE: a simulation study. Molecular Ecology.

[b17] Excoffier L, Smouse PE (1994). Using allele frequencies and geographic subdivision to reconstruct gene trees within a species – molecular variance parsimony. Genetics.

[b18] Falush D, Stephens M, Pritchard JK (2003). Inference of population structure using multilocus genotype data: linked loci and correlated allele frequencies. Genetics.

[b19] Gandon S, Day T (2009). Evolutionary epidemiology and the dynamics of adaptation. Evolution.

[b20] Garrett KA, Mundt CC (2000). Host diversity can reduce potato late blight severity for focal and general patterns of primary inoculum. Phytopathology.

[b21] Goyeau H, Halkett F, Zapater MF, Carlier J, Lannou C (2007). Clonality and host selection in the wheat pathogenic fungus *Puccinia triticina*. Fungal Genetics and Biology.

[b22] Halkett F, Simon JC, Balloux F (2005). Tackling the population genetics of clonal and partially clonal organisms. Trends in Ecology and Evolution.

[b23] Haverkort AJ, Boonekamp PM, Hutten R, Jacobsen E, Lotz LAP, Kessel GJT, Visser RGF (2008). Societal costs of late blight in potato and prospects of durable resistance through cisgenic modification. Potato Research.

[b24] Kawecki TJ, Ebert D (2004). Conceptual issues in local adaptation. Ecology Letters.

[b25] Kiyosawa S, Purba D, Ali MS, Okinaka Y, Shimizu T, Saito A (1995). Influence of frequencies of host genotypes and fitness of virulence genes on nonrandom association type and pattern between avirulence loci. Breeding Science.

[b26] Knapova G, Gisi U (2002). Phenotypic and genotypic structure of *Phytophthora infestans* populations on potato and tomato in France and Switzerland. Plant Pathology.

[b27] Langella O (1999). Populations 1.2.32, Population Genetic Software.

[b28] Lannou C (2012). Variation and selection of quantitative traits in plant pathogens. Annual Review of Phytopathology.

[b29] Leach JE, Vera-Cruz CM, Bai JF, Leung H (2001). Pathogen fitness penalty as a predictor of durability of disease resistance genes. Annual Review of Phytopathology.

[b30] Lees AK, Wattier R, Shaw DS, Sullivan L, Williams NA, Cooke DEL (2006). Novel microsatellite markers for the analysis of *Phytophthora infestans* populations. Plant Pathology.

[b31] Leonard KJ (1994). Stability of equilibria in a gene-for-gene coevolution model of host-parasite interactions. Phytopathology.

[b32] Montarry J, Glais I, Corbière R, Andrivon D (2008). Adaptation to the most abundant host genotype in an agricultural plant–pathogen system – potato late blight. Journal of Evolutionary Biology.

[b33] Montarry J, Hamelin FM, Glais I, Corbière R, Andrivon D (2010a). Fitness costs associated with unnecessary virulence factors and life history traits: evolutionary insights from the potato late blight pathogen *Phytophthora infestans*. BMC Evolutionary Biology.

[b34] Montarry J, Andrivon D, Glais I, Corbière R, Mialdea G, Delmotte F (2010b). Microsatellite markers reveal two admixed genetic groups and an ongoing displacement within the French population of the invasive plant pathogen *Phytophthora infestans*. Molecular Ecology.

[b75] Nei M (1978). Estimation of average heterozygosity and genetic distance from a small number of individuals. Genetics.

[b35] Nesse RM (2011). Ten questions for evolutionary studies of disease vulnerability. Evolutionary Applications.

[b36] Nesse RM, Bergstrom CT, Ellison PT, Flier JS, Gluckman P, Govindaraju DR, Niethammer D (2010). Making evolutionary biology a basic science for medicine. Proceedings of the National Academy of Sciences of the United States of America.

[b37] Pariaud B, Ravigné V, Halkett F, Carlier J, Lannou C (2009). Aggressiveness and its role in the adaptation of plant pathogens. Plant Pathology.

[b38] Philips SL, Shaw MW, Wolfe MS (2005). The effect of potato variety mixtures on epidemics of late blight in relation to plot size and level of resistance. The Annals of Applied Biology.

[b39] Pilet F, Chacón M, Forbes GA, Andrivon D (2006). Protection of susceptible potato cultivars against late blight in mixtures increases with decreasing inoculum pressure. Phytopathology.

[b40] Pritchard JK, Stephens M, Donnelly P (2000). Inference of population structure using multilocus genotype data. Genetics.

[b71] R Foundation for Statistical Computing (2012).

[b41] Raymond M, Rousset F (1995). Genepop Version1.2: population genetics software for exact tests and ecumenicism. Journal of Heredity.

[b42] Rousset F (1997). Genetic differentiation and estimation of gene flow from F-statistics under isolation by distance. Genetics.

[b43] Skelsey P, Rossing WAH, Kessel GJT, Werf van der (2010). Invasion of *Phytophthora infestans* at the landscape level: how do spatial scale and weather modulate the consequences of spatial heterogeneity in host resistance?. Phytopathology.

[b44] Slatkin M (1995). A measure of population subdivision based on microsatellite allele frequencies. Genetics.

[b45] Spielman LJ, Drenth A, Davidse LC, Sujkowski LJ, Gu W, Pooley PW, Fry WE (1991). A second worldwide migration and population displacement of *Phytophthora infestans*. Plant Pathology.

[b46] Tellier A, Brown JKM (2011). Spatial heterogeneity, frequency-dependent selection and polymorphism in host-parasite interactions. BMC Evolutionary Biology.

[b47] Thrall PH, Burdon JJ, Bever JD (2002). Local adaptation in the *Linum marginale**Melampsora lini* host-pathogen interaction. Evolution.

[b48] Weir BS, Cockerham CC (1984). Estimating F-statistics for the analysis of population structure. Evolution.

[b49] Whitlock MC (2008). Evolutionary inference from QST. Molecular Ecology.

[b50] Whitlock MC, Gilbert KJ (2012). QST in a hierarchically structured population. Molecular Ecology Resources.

